# Sleep, nutritional status and eating behavior in children: a review study

**DOI:** 10.1590/1984-0462/2022/40/2020479IN

**Published:** 2022-09-09

**Authors:** Fernanda Nascimento Hermes, Eryclis Eduardo Miguel Nunes, Camila Maria de Melo

**Affiliations:** a Universidade Federal de Lavras, Lavras, Minas Gerais, Brazil.

**Keywords:** Sleep, Nutritional status, Physical activity, Child, Sono, Estado nutricional, Atividade física, Criança

## Abstract

**Objective:**

To review the current literature on the relationship between sleep, nutritional status and eating behavior, as well as mechanisms associated with these elements in children.

**Data source:**

The literature research was conducted in the PubMed, LILACS and Scopus databases, using the following terms: “Child”; “Nutritional status”; “Sleep”; “Physical activity OR Physical activities OR Exercise”. The articles included were those that met the research objective. Review articles, letters to authors, or guidelines were excluded.

**Data synthesis:**

402 articles were initially found in the literature search. After careful analyses of the title and abstract, and application of inclusion criteria, only 24 studies were included in the present review. Most studies (n=13) suggest that short sleep duration (<9-10 hours/night) is associated with overweight/obesity in children. Only three studies did not show associations between overweight/obesity and sleep variables. Short sleep duration is also associated with poor food quality, higher intake of soft drinks and stimulant beverages before bedtime, as well as micronutrient deficiency.

**Conclusions:**

Sleep duration is related to overweight and obesity development in infants. Changes in dietary pattern are also related to sleep debt, being one of the mechanisms that contribute to excessive weight gain. It is necessary that health professionals understand the importance of sleep quality in the nutritional status maintenance in children.

## RESUMO

**Objetivo:**

Fazer uma revisão da literatura sobre a relação entre sono, estado nutricional e padrão alimentar, bem como mecanismos associados, na população infantil.

**Fontes de dados:**

Foram realizadas buscas bibliográficas nas bases PubMed, LILACS e Scopus, usando os seguintes descritores: “*Child*”; “*Nutritional status*”; “*Sleep*”; “*Physical activity OR Physical activities OR Exercise*”. Os artigos que atenderam ao objetivo da pesquisa foram incluídos. Excluíram-se estudos de revisão, cartas para autores ou diretrizes.

**Síntese dos dados:**

Inicialmente, 402 artigos foram encontrados na pesquisa. Após análise dos títulos e abstracts e a aplicação dos critérios de exclusão, 24 estudos foram selecionados para a revisão. A maioria dos estudos (n=13) sugere que sono de curta duração (<9-10 horas/noite) está associado com sobrepeso/obesidade na população infantil. Apenas três artigos não reportaram associações entre variáveis do sono e estado nutricional. Curta duração do sono também foi associada a hábitos alimentares ruins, maior consumo de refrigerantes e bebidas estimulantes antes de deitar, bem como deficiência de micronutrientes.

**Conclusões:**

A duração do sono tem relação com sobrepeso e obesidade na população infantil. Alterações no padrão alimentar também têm relação com débito de sono, sendo um dos mecanismos que contribuem para o ganho de peso excessivo. É necessário que os profissionais de saúde tomem conhecimento da importância da qualidade do sono para a manutenção do estado nutricional em crianças.

## INTRODUCTION

Obesity is a global epidemic that has been affecting all strata of the population, including children and adolescents. The number of overweight/obesity cases among the younger population has grown about ten times in the last four decades, corresponding to around 50 million girls and 74 million boys worldwide.^1^ A survey conducted in Brazil by the World Health Organization (WHO), in 2016, showed that about 41 million children under five years of age were overweight.^2^

The nutritional status of children observed nowadays is the result of changes made in people’s lifestyle in recent decades, especially in young people, with the increased consumption of ultra-processed food, fast-food, and sugar-rich food, associated with a predominance of sedentary activities and the excessive use of electronic equipment.^2^ In addition to these factors, sleep quality has been associated with nutritional status.^3^

Several studies have shown the association between sleep duration and the development of obesity in adults and children^3^. Although the mechanisms involved are uncertain, some possible pathways might explain this causal relationship, including changes in hormonal production, physical activity level, and eating behavior.^3^ ^,^ ^4^ ^,^ ^5^

The sleep debt might influence both sides of energy balance, resulting in increased energy intake and lower energy expenditure. As a consequence of fatigue caused by sleep loss, lower energy expenditure during the day is observed, which leads to a sedentary behavior.^6^ In addiction, higher energy intake and higher intake of palatable foods, such as sugar and fat-rich food, are seen in response to sleep debt.^7^ In adults, sleep restriction has been associated with changes in hormonal regulators of energy balance, such as increased ghrelin and decreased leptin secretion.^8^ In children, short sleep duration has been associated with higher energy intake in early life^9^ and hedonic eating.^10^ Also, insulin resistance and increased cortisol levels are seen in association with sleep debt.^11^ The insulin resistance might alter lipid profile and increase metabolic risk in children and adolescents, especially when associated with overweight and physical inactivity.^12^

Thus, this study aims to review the literature about the relationship between sleep and nutritional status in children, as well as discuss the mechanisms associated with this relationship in this population.

## METHOD

The present study represents an integrative review. The guiding question was: what is the relationship between sleep deprivation, nutritional status, and physical activity level in children? This research question was based in PI(E)COS formulation, in which: Population=children; Intervention or exposure=sleep duration; Comparison=any; Outcomes=nutritional status. The steps taken to construct the research are described below.

Bibliographic research was carried out in the PubMed, LILACS and Scopus databases, using the descriptors indicated by Medical Subject Headings (MeSH): “Child”; “Nutritional status”; “Sleep”; “Physical activity OR Physical activities OR Exercise”. Articles in Portuguese, English and Spanish, published until 2019, were included.

Reviewed studies were selected according to the following inclusion criteria:

1. Children population;
2. Non-inclusion of subjects with any sleep disorder (e.g., obstructive sleep apnea, insomnia, restless legs syndrome, etc.) or who were using appetite-suppressing medications;
3. Evaluation of the relationship between sleep and nutritional status, dietary pattern or level of physical activity. Review articles (systematic, integrative, narrative) and letters to authors were excluded.

In order to select the studies, the following steps were taken: first, the title and abstracts were analyzed and articles that didn’t match our inclusion criteria were excluded; second, the selected articles were read in full to identify possible exclusion criteria and duplicated studies; and third, data was extracted using a Microsoft Excel spreadsheet, containing information such as authors’ names, article title, published journal and year of publication, objective, keywords and main results.

Quality analysis was performed using the Quality Assessment Tool for Observational Cohort and Cross-Sectional Studies from the National Institutes of Health (NIH).^13^ Also, the compliance of each study was checked by the Strengthening the Reporting of Observational Studies in Epidemiology association (STROBE).^14^

## RESULTS

**Figure 1:**
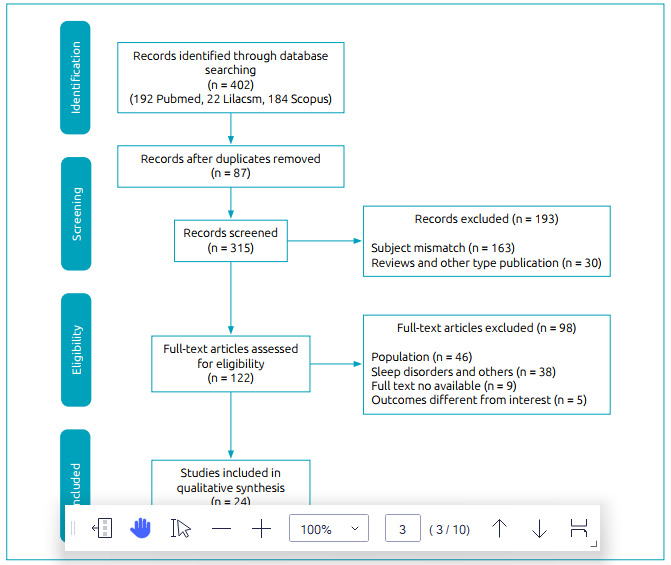
Flowchart of study selection.

As shown in Figure 1, 402 articles were initially found in the literature search: 196 from the PubMed database, 22 from the LILACS database, and 184 from the Scopus database. After screening by title and abstract, 193 articles were excluded for not attending to our research question. After reading full-text articles, 98 were excluded for not attending to the inclusion criteria, 87 articles were excluded as duplicated, and two were not found in full. Therefore, only 23 articles were included in the present review.

The analyzed studies were published between 2002 and 2019; 22 of them were cross-sectional and one of them was longitudinal. The majority of analyzed studies (n=13) suggests that short sleep duration (<9-10 hours/night) is associated with overweight/obesity and nutritional status in the infant population; 8 of them (34.7%) point out that sleep duration is related to eating behavior.

Sleep was objectively evaluated by polysomnography in only one study;^15^ all others used questionnaires to assess sleep duration and quality. The questionnaires used were either developed by the authors (n=16) or validated sleep questionnaires, such as the Pediatric Sleep Questionnaire (PSQI; n=3), the Children Sleep Habit Questionnaire (CSHQ; n=1), the Sleep Disturbance Scale for Children (SDSC; n=1) and the Brief Infant Sleep Questionnaire (BISQ; n=1). The mean age of the studied population ranged from 6 months to 18 years. Tables 1, 2 and 3 present a brief description of the included studies.

**Table 1:** Description and main results of the included studies with children between six months and eight years old.

Author (year)	Goals and Sample	Main results
King (2017)^7^	To analyze the association between soft drink consumption and child behavior, according to food safety status and sleep patterns; 2,829 children; ±5 years old.	Sleep problems were associated with soft drink intake in food-insecure children.
Carrillo-Larco et al. (2014)^16^	To describe nutritional status and sleep duration in children from four countries; >8,000 children; ±8 years old.	Sleep duration <10h was associated with 15% increased incidence of obesity. Maternal education and family income attenuated this relationship.
Na et al. (2019)^32^	To examine the association between food insecurity and child sleep outcomes; 362 preschoolers; 7-8 years old.	Child food insecurity was associated with a 2.25 times increase in poor child sleep quality in the adjusted model.
Ruotolo et al. (2016)^33^	To verify whether nocturnal eating habits can influence sleep and parasomnia in children; 226 children; 7-8 years old.	45% of children who presented parasomnia during the night consumed stimulant caffeine-rich food before bedtime. Late dinner was associated with late bedtime.
Kordas et al. (2007)^34^	To investigate the relationship between lead exposure, micronutrient status, sleep, classroom behavior and physical activity in Mexican children; 602 children; 6-8 years old.	Blood lead≥10μg/dL was associated with later waking time and shorter duration of sleep. Anemia was linked to earlier bedtime, and shorter sleep onset.
Kordas et al. (2008)^35^	To investigate the relationship between iron deficiency anemia (ADI), swimming and maternal sleep reports; 1,270 children; 6-18 months of age.	Iron deficiency, low length and stunting were associated short sleep duration and higher frequency of night wakening; stunting was related to shorter nap duration.
Durán et.al. (2012)^37^	To determine the association of sleep duration and obesity; 155 schoolchildren; 5-7 years old.	Sleeping >10 hours/night, exercising, and not eating chocolate at night are associated factors for obesity.

**Table 2:** Description and main results of the included studies with children between six and ten years old.

Author (year)	Goals and sample	Main results
Marques et al. (2018)^6^	To analyze the association between sleep duration and nutritional status of school-age children studying in Portugal; 829 students; ±9 years old.	There was a weak but significant correlation between sleep duration and body mass index z-score (r=0.15; p<0.01).
Agüero et al. (2017)^19^	To establish a relationship between sleep duration, nutritional status and consumption patterns of caffeinated beverages; 805 school-age children; 6-10 years old.	Normal weight (NW) subjects slept significantly more hours than obese participants (9.8±0.9 vs. 9.6±0.9). Sleep duration during the week was inversely associated to obesity (OR 3.5, 95%CI 1.3-9.2).
Chamorro et al. (2014)^15^	To compare the characteristics of sleep microstructure (CAP) in overweight (OW) and normal weight children; 58 children; 10 years old.	CAP time and CAP rate showed significant associations with body mass index z-score. Obese subjects might have less stable slow wave sleep episodes.
Corso et al. (2012)^24^	To analyze the association between OW/obesity (OB) and hours spent with TV/computer, hours of sleep and physical activity; 4,964 schoolchildren; 6-10 years old.	OW and OB were associated with weekly sports practice and sleep hours per night.

**Table 3:** Description and main results of the included studies with children and adolescents.

Author (year)	Goals and Sample	Main results
Agüero and Rivera (2016)^17^	To investigate sleep and eating habits, physical activity and nutritional status; 1,810 children; 6-11 years old.	Positive associations between sleep and risk of being obese, even when adjusted for confounders.
Bazán et al. (2018)^18^	To describe factors associated with overweight (OW)/obesity (OB); 3,752 children; 2-15 years old.	OB was higher in children who sleep less than the recommended hours of sleep.
Cicek et al. (2009)^20^	To examine the risk factors associated with arm fat area (AFA) in Turkish children and adolescents; 5,358 schoolchildren; 6-17 years old.	Sleeping 8-9 h and ≤8 h significantly predicted OW for boys; while sleeping 9-10 h, 8-9 h and ≤8 h significantly predicted OW for girls.
Katsa et al. (2018)^22^	To investigate the effect of life habits on Child Metabolic Syndrome; 480 children; 5-12 years old.	Late bedtime was associated with increased weight, waist circumference, lower height and blood glucose.
Guo et al. (2012)^23^	To compare health-related factors among Chinese children and adolescents with normal weight (NW), OW and OB; 4,262 children and adolescents; 5-18 years old.	NW children had a higher change of having a longer sleep duration than OB. OB children were less likely to sleep longer (≥7.5 h) than the NW.
Jong et al. (2012)^26^	To establish an association between sleep duration and OW in childhood; 4,072 children; 4-13 years old.	Short sleep duration was associated with watching TV during meals and eating sugar-rich foods.
Aravena et al. (2017)^28^	To study nutritional status and sleep duration in Chilean children; 481 schoolchildren; 6-15 years old.	The numbers for OB children were not different from those for NW children who sleep the recommended hours.
Giovaninni et al. (2014)^29^	To analyze clock gene polymorphism and presence of OB and sleep duration; 370 children; 6-13 years old.	Trend toward higher prevalence of OW in children <9h/night of sleep vs >10h/night.
Golley et al. (2013)^30^	To investigate if sleep time is associated with diet; 2,200 children and adolescents; 9-16 years old.	Late bedtime group had higher body mass index (BMI); there were significant associations between BMI, sleep duration and energy intake.
Jiang et al. (2014)^21^	To investigate the association between sleep duration and somatic growth; 143 children; 10-11 years old.	Children that slept >10h/night had higher weight, height and BMI.
Christoph et al. (2017)^27^	To investigate weight-related factors in rural and urban schoolchildren; 148 schoolchildren; 11-16 years old.	BMI was positively related to being female, rural, more active and having higher subjective sleep quality.
Hitze et al. (2009)^31^	To investigate the determinants of sleep duration in children; 414 adolescents; 13 years old.	Short vs long sleep was associated with 5.5-/2.3-fold higher risks for OB in girls.

From our search, 16 studies investigated the relationship between nutritional status and sleep pattern. Most of the studies found some association between nutritional status and sleep loss. On average, sleeping less than 10-9 hours was associated with overweight/obesity or a higher incidence of obesity was found in short-sleepers.^6^ ^,^ ^15^ ^,^ ^16^ ^,^ ^17^ ^,^ ^18^ ^,^ ^19^ ^,^ ^20^ ^,^ ^21^ ^,^ ^22^ ^,^ ^23^ ^,^ ^24^ ^,^ ^25^ ^,^ ^26^

In a cross-sectional study conducted with 1,810 children aged between 6 and 11 years, sleep quality was evaluated by the Pittsburgh Sleep Quality Index (PSQI) questionnaire, and the results showed that 49.9% of the evaluated children slept less than recommended (<10 hours a day), and this sleep restriction was higher during weekdays and among older children. Shorter sleep time was considered a risk factor for overweight/obesity development.^17^

In Brazil, a cross-sectional study with 4,964 elementary school students indicated the prevalence of 15.4% of overweight and 6.0% of obesity, which was associated with reduced sports practice, shorter sleep duration/night, and longer time using the computer.^24^

Only Chamorro et al.^15^ used the gold standard method of polysomnography to evaluate sleep. The study compared sleep structure in normal and overweight ten year old children. The authors observed that there was an inverse relationship between body mass index (BMI) and sleep duration, and overweight children showed reduced sleep duration, efficiency, and an atypical sleep cycle during the night, with reduced stage 3 non-rapid eye movement (NREM) sleep at the beginning of the night, and increased stage 3 NREM sleep at late night.

Other anthropometrical parameters besides body weight and BMI were associated with sleep duration. Cicek et al.,^20^ in a sample of 5,358 children and adolescents from 6 to 17 years old, found a positive association between arm fat area and sleep duration below eight hours.

Katsa et al.,^22^ in a study that investigated life habits and predisposition to metabolic syndrome in children between 5 and 12 years old, found that late bedtime is positively related to weight, lower length, and waist circumference. They also found associations between late bedtime, blood glucose and diastolic blood pressure.

In contrast, Christoph et al.^27^, in a study in Africa, with 146 adolescents between 11 and 16 years old, showed that BMI was positively associated with sleep quality. They also found a positive association between BMI and being more physically active. Aravena et al.^28^ failed to find an association between sleep and nutritional status. However, they found that, during weekdays, the percentage of obese children who sleep the recommended hours was 69.2%, less than that of overweight children (73.5%) and children with normal weight (75%) (p=0.215). On weekend days, the percentage of normal weight children that slept the recommended hours was 63%, while that percentage was 65.7% in the obese group and 68.4% in the overweight group (p=0.781). Also, Giovaninni et al.^29^, in a cross-sectional study with 370 children and adolescents between 6 and 13 years, found that sleep duration was inversely related to age (p<0.001), but found only a trend toward a higher prevalence of overweight in children who slept less than nine hours (23%) when compared to those who slept more than ten hours (16%, p=0.06).

Regarding food patterns, we found eight studies that investigated associations between sleep and food habits or behavior. Fewer hours of sleep and late bedtime have shown to be related to a poor quality diet.

Golley et al.,^30^ in a study with 2,200 Australian children, concluded that early bedtime is associated with better diet quality and higher intake of fruits and vegetables, and that those who showed a late bedtime presented high consumption of poor quality food.

Higher intake of fast-food and soft drinks was observed in children with short sleep duration (<9 hours/night) in the Kiel Obesity Prevention Study.^31^ King et al.^7^ also showed a higher intake of soft drinks in children under food insecurity with sleep difficulties. In the study of Na et al.,^32^ food insecurity was associated with poor sleep quality. Jong et al.^26^ investigated some determinants of short sleep duration. The eating habits associated with short sleep duration varied across ages and sex, but, overall, eating sweetened food, a lack of an eating routine and watching TV during the meal was associated with short sleep duration.

The consumption of stimulant drinks, such as chocolate milk, soft drinks, coffee and black tea at night contribute to a 2.6 higher risk for sleep disturbance and influence sleep quality in children between seven and eight years old.^34^ Micronutrient deficiency also showed a relation to sleep patterns. In the studies done by Kordas et al.,^34^ ^,^ ^35^ iron-deficient children showed sleep disruptions, such as early wake up, increased sleep latency, sleep fragmentation, and reduction in sleep duration.

The quality of the studies included in our review was checked by the NIH tool.^13^ The studies of Guo et al.^23^ and Durán et al.^25^ had the lowest score in the quality analysis, and were considered poor, with six positive answers (in a total of 14) in the NIH tool. Most of the studies were considered fair and had between seven and nine positive answers in the NIH tool (King et al.,^7^ Kordas et al.,^34^ Christoph et al.,^27^ Katsa et al.,^22^ Carrillo-Larco et al.,^16^ Kordas et al.,^35^ Jiang et al.,^21^ Jong et al.,^26^ Hitze et al.,^31^ Chamorro et al.,^15^ Ruotolo et al.,^33^ Cicek et al.,^20^ Agüero et al.,^19^ Durán et al.,^25^ Corso et al.,^24^ Bazán et al.,^18^ Marques et al.,^6^ Aravena et al.,^28^ Giovaninni et al.^29^), and the highest quality studies were the ones made by Agüero and Riveira,^17^ Golley et al.^30^ and Na et al.,^32^ with ten positive answers. The compliance with the STROBE checklist showed that only three studies fulfilled more than 75% (Agüero et al.,^19^ Chamorro et al.^15^ and King et al.^7^) of the items of the checklist, and the others accomplished between 43.7 and 71.8%. The main issues found were an inadequate description of sample size and efforts to address the potential source of bias, treatment of missing data, discussion of limitations and generalizability of the study results.

## DISCUSSION

Sleep is defined as the moment of complete rest in which there is a decrease in consciousness, reduction of muscle movements, and slowing of the body. It has a restorative function and is the time when memory consolidation occurs. In addition, it plays a critical role in brain functions, such as mood regulation and cognitive performance, and is critically involved in regulating metabolism, acting in appetite control, immunological functions, among others.^36^

Sleep is classified into two different phases. NREM (non-rapid eye movement) sleep is when physical rest occurs, and is related to the immune system and the rhythm of the digestive system function. Throughout NREM sleep, three sleep stages are passed, progressing from more superficial sleep to slow-wave sleep, a deeper sleep stage. In the NREM sleep stage 1, there is a transition from the waking state to sleep; this is the lightest stage of sleep. In the second phase, NREM sleep stage 2, eye movements stop and deepest sleep begins. In the third and final phase, NREM sleep stage 3, known as slow-wave sleep or deep sleep, awakening becomes more difficult, there is a decrease in muscle tone and hormonal secretion, such as growth hormone (GH).^36^ ^,^ ^37^

In REM (rapid eye movement) sleep, psychological rest, emotional well-being, and memory consolidation occurs. REM sleep is also known as the dream phase, due to increased brain activity. At this stage, there is a decrease in muscle tone, increased heart rate, and irregular breathing.^37^ ^,^ ^38^ ^,^ ^39^

During the first year of life, there are several changes in the sleep architecture until NREM and REM sleep stages are consolidated. During pregnancy, the fetus is influenced by maternal circadian rhythm, and, after birth, a period of adaptation is required until sleep-wake cycle is established.^40^

In the first three months of life, a newborn sleeps about 16 to 18 hours a day. In this stage of life, three phases of sleep are seen: quiet sleep (similar to NREM sleep), active sleep (similar to REM sleep), and indeterminate sleep. The sleep onset occurs in REM sleep, and each episode consists of only 1 or 2 cycles, which is the result of the lack of establishment of the circadian rhythm.^41^

After three months of age, waking time during the day increases and longer periods of sleep at night are observed, due to the beginning of hormonal cycling of cortisol and melatonin. These hormones are responsible for circadian rhythm control regulated by environmental stimuli (breastfeeding routines and bedtime). At this stage, the sleep cycles of NREM and REM become more regular.^41^

At 12 months of age, the child sleeps about 12 to 16 hours a day, with most of the sleep consolidated in the night and some naps during the day. Studies suggest that children spend more time in REM sleep when compared to adolescents.^41^ ^,^ ^42^

Over the years, the sleep duration seems to decrease in the child population in several countries. Between 1905 and 2008, there was an average reduction of 75 minutes in sleep duration in this population.^43^ Changes in sleep patterns were accompanied by lifestyle changes, such as the use of electronic equipment (tablets, mobile phones, computers, television, etc.). The overuse of this equipment has been related to sleep duration and quality.^44^

Studies suggest that the presence of screens in the bedroom is associated with a decrease in sleep time, due to a greater propensity to use these equipments.^45^ A study conducted in the United States, with 48,687 children of the National Survey of Children’s Health, showed that the presence of a television in the bedroom was associated with insufficient sleep for more than four nights per week.^46^ The use of television during meals also had a negative effect on sleep duration in a cross-sectional study with 4,072 children, aged between 4 to 13 years, in the Netherlands.^26^ Also, the sedentary behavior resulting from the excessive use of electronic equipment favors weight gain, in addition to the negative effect on sleep quality.^47^

Several studies report an association between overweight/obesity and the excessive use of electronic equipment as a reflection of sedentary lifestyle.^24^ ^,^ ^18^ A study carried out in Spain with 3,752 individuals aged from 2 to 15 years old showed that children who spent more than two hours/day using electronic devices had higher rates of overweight when compared to those who spent less than two hours/day in these activities.^16^

One possible causal effect between the use of electronic devices and reducing sleep is the suppression of melatonin.^44^ Several neurotransmitters and hormones control the wake-sleep cycle. Melatonin is a hormone primarily secreted by the pineal gland in the absence of light, and induces sleepness.^36^ The blue light emitted by electronic equipment, especially at night, may result in decreased melatonin release and consequent delay of sleep onset. This event may lead to circadian cycle deregulation, thus decreasing duration and quality of sleep.^44^ ^,^ ^48^ This becomes a vicious circle in the child’s routine, since excessive exposure to light emitted by these equipment affects sleep quality, and insufficient sleep predisposes to the use of such equipment.^32^

Sleep debt might result in several deleterious consequences for health, such as stress, physical fatigue, difficulty concentrating, among others.^19^ ^,^ ^25^ Metabolic and behavior changes have also been related to sleep loss, which contributes to overweight and obesity.^16^ ^,^ ^17^ ^,^ ^27^ ^,^ ^19^ ^,^ ^25^ Out of the 16 studies included in our literature search, 14 showed positive associations between short sleep duration and the development of overweight in the infant population. Three studies had a negative association between sleep and overweight/obesity.

Moreover, sleep loss may predispose to weight gain due to deregulation of metabolic profile during the night, such as insulin metabolism, increased cortisol secretion and decreased growth hormone (GH) concentration, which may favor lipogenesis.^31^ ^,^ ^49^

Among the mechanisms behind the relationship between sleep loss and overweight, food intake is influenced by sleep patterns and can trigger overweight/obesity.

The main hypothesis to explain this relationship is the fact that individuals with sleep restriction remain longer in wakefulness, which consequently increases energy demand, causing an increase in caloric intake in periods of sleep loss.^10^ Also, a greater propensity to hedonic eating among individuals with short sleep duration can lead to higher caloric intake in this population. In a cross-sectional study from the New Zealand twin cohort, it was observed that children who slept less at five years of age were more likely to have food responsiveness, i.e., these children had a higher preference for highly palatable and consequently more caloric foods, characterizing hedonic and non-physiological hunger.^50^ ^,^ ^51^

In association with this, studies report a higher prevalence of the consumption of food high in energy density and poor in nutrients in children who sleep later. These children showed a preference for fast-food, pastries, sweetened beverages, soft drinks, and a lower consumption of fruits and vegetables.^7^ ^,^ ^30^ ^,^ ^31^ Likewise, the high consumption of stimulant foods, such as chocolate and soft drinks, may be associated with the development of sleep disorders, such as parasomnias.^33^

These changes in dietary patterns, with the increase in low nutritional quality food consumption, lead to micronutrient deficiency, such as anemia, due to the low consumption of iron-rich foods. It’s important to highlight the relationship of anemia with reductions in sleep duration and physical activity level.^34^ ^,^ ^35^

Studies conducted in adults suggest that, in sleep loss conditions, there is a change in the appetite-regulating hormones, causing a decrease in leptin and an increase in ghrelin concentrations, causing a greater sense of hunger.^8^ However, a study conducted with 414 individuals, aged between 6 and 19 years old, observed an increase in leptin concentrations in girls with sleep restriction, which suggests that chronic sleep loss may lead to future changes in metabolism.^31^

However, it’s important to emphasize that there is still a limited number of studies that try to explain mechanisms related to sleep loss, metabolic consequences and weight gain. Also, a frequent problem is the lack of studies with objective measurements of sleep, as most studies do not use validated questionnaires to address sleep duration and quality. Moreover, studies with careful evaluation and analysis of food intake and behavior, in addition to a valid measurement of sleep in this population, are needed. It is important to emphasize that the present literature review was conducted with a specific population (children), and the conclusions might be different across other population strata. Also, the proposed mechanisms to explain sleep and nutritional status relationship come from studies in adults or experimental studies. This reinforces the importance of studying these mechanisms in infant population.

It is possible to conclude, based on the reviewed literature, that sleep duration is related to the development of overweight and obesity in the infant population. The amount of sleep also contributes to changes in eating behavior, being one of the contributing mechanisms to weight gain. Therefore, health professionals must recognize the association between lifestyle parameters, such as sleep, eating behavior, and physical activity level, and be able to include sleep recommendations in nutritional counseling.
